# Birds and environment: a multidisciplinary approach to ecological, behavioural and conservation issues

**DOI:** 10.1186/s40850-024-00199-8

**Published:** 2024-04-28

**Authors:** Ashish Kumar Arya, Archana Bachheti, Vinaya Kumar Sethi, Kamal Kant Joshi

**Affiliations:** 1 Department of Environmental Science, Graphic Era (Deemed to be University), Dehradun, 248002 Uttarakhand India; 2 Uttarakhand Sanskrit University, Haridwar, India; 3 Department of Environmental Science, Graphic Era Hill University, Dehradun, 248002 Uttarakhand India

## Abstract

Birds perform significant ecosystem services in the environment. Nevertheless, they have been facing threats to their survival globally. This special collection assembles diverse articles on various aspects of birds’ life, their interactions with the environment, their adaptations, and threats they have been facing along with conservation measures.

## Main text

Birds are found in most habitats across the globe. They are regarded as an integral component of the ecosystem, where they play key roles in a variety of natural processes such as seed dispersal, predation, pollination, scavenging and ecosystem engineering.

Seed dispersal is a crucial phenomenon affecting population dynamics, community structure and biodiversity of an area. Birds such as hornbills, bulbuls and jays have been regarded as expert seed dispersers due to their ability to cover much longer distances when compared with other means of seed dispersal such as wind. Also, they assist in the regeneration of forests and plants in general, through connecting different habitats [1]. Similarly, birds such as sunbirds and hummingbirds are known as important pollinators in their ecosystems.

The roles of birds in nutrient transfer and soil formation have also been well studied across various habitats. For example, the key role of seabirds in cycling nutrients and fertilising marine ecosystems such as coral reefs is a well-cited example. Additionally, birds such as vultures act as essential scavengers by removing the carcases of dead animals and providing natural sanitation services.

Furthermore, the contribution of birds in controlling pest populations is well recognized. Birds add to preserve a delicate balance in agricultural settings through feeding on insects, which pose a substantial threat to crops. In addition to providing an excellent solution to pest management in agricultural systems, birds reduce the risk of insect-borne diseases. The significant involvement of birds in pest control moves beyond invertebrate pests, as several birds also feed on rodent pests as a major portion of their diet [2]. Therefore, a reduction of bird populations might lead to an increase in pest populations, an increased need for the use of chemical pesticides, endangering crop yield and ultimately affecting global food security.

Birds’ sensitivity to factors such as habitat loss and climate change has helped scientists to better understand the impacts of human activities on the natural world [3, 4]. According to the popular study entitled ‘The Millennium Ecosystem Assessment’, there are four major categories of ecosystem services, i.e. provisioning, regulating, cultural and supportive services, for the benefits of mankind [5] and interestingly birds contribute to all four of these services [6].

Birds face numerous challenges caused by human activities, resulting in population declines worldwide due to habitat degradation, poaching, pollution, climate change, poisoning, collision with wind turbines or buildings and pesticide usage [7].

Therefore, it is critical to conserve birds and preserve their crucial services through addressing these concerns and taking appropriate steps to reduce disastrous human impacts on their fragile ecosystems. To maintain bird biodiversity and assure their continuing involvement in environmental balance, comprehensive conservation initiatives are essential. These may include protecting and restoring natural habitats, implementing sustainable farming practices, continuous monitoring of birds, and increasing awareness of birds’ role in the ecosystem [8]. Furthermore, international collaboration is critical for solving global concerns.

In conclusion, birds play a crucial role in a large number of ecosystem services and, therefore, effective measures should be taken to conserve their habitats. In this Collection, we explore anthropogenic factors that affect their natural environment, behaviours, migration patterns, and population patterns worldwide. We invite authors to contribute their observations on birds and their changing environment in order to enable better conservation strategies in the future.

## Data Availability

Not applicable.
